# One Model, Many Cities: A Transferable Social Relationship Inference Framework for Human Mobility Data

**DOI:** 10.1145/3748636.3762710

**Published:** 2025-12-12

**Authors:** Chen Chu, Cyrus Shahabi, Emmanuel Tung, Khurram Shafique

**Affiliations:** University of Southern California, Los Angeles, California, USA; University of Southern California, Los Angeles, California, USA; Novateur Research Solutions, Ashburn, Virginia, USA; Novateur Research Solutions, Ashburn, Virginia, USA

**Keywords:** Social Relationship Inference, Human Mobility Modeling, Embedding Alignment, Transfer Learning, Domain Adaptation, Information systems, Geographic information systems

## Abstract

Inferring social relationships from mobility data is crucial for many applications because it reflects real-world connections among people. However, large-scale trajectory datasets with ground-truth social ties are exceedingly scarce, making it difficult to train deep models for relationship inference. To address this gap, we propose a transferable social relationship inference framework that can be trained on one high-quality, labeled dataset and then generalized to new datasets, even from different cities.

Our framework rests on the key insight that social bonds depend largely on the frequency of individual meetings and the popularity of those meeting locations, both of which can be inferred statistically from raw trajectory data, irrespective of the underlying geographic semantics. It comprises two main modules: 1) Universal Social Relationship Classifier (USRC): A model trained to infer social relationships from trajectory data, and 2) Spatial Embedding Transfer (SET): A location embedding alignment technique that adapts new datasets to the pre-trained USRC model.

By aligning location embeddings, SET module enables the pre-trained USRC to interpret previously unseen datasets without extra supervision. Experiments on five public datasets demonstrate that our method achieves state-of-the-art performance in zero-shot social relationship inference, surpassing other unsupervised, and in some cases, even supervised, approaches. Additionally, the SET module significantly improves location embedding alignment, outperforming existing baseline methods. The source code and data are available at https://github.com/chuchen2017/SET.

## 1 Introduction

Individuals’ social activities greatly influence their mobility behaviors, for example, friends often visit the same places together [6, 8, 10, 33]. As previous studies show [3, 16, 24–26], social ties between users can be accurately inferred using mobility data. Inferring social relationships from mobility data is critical because it captures the physical connections people form in the real world. This knowledge not only supports traditional online social network applications, such as targeted advertising, recommendation systems, and social or cultural studies, but also enables unique use cases. For example, they can help identify unknown members of criminal or terrorist organizations or model disease transmission through human contact in epidemiology. Additionally, they have been studied in the context of location privacy, where social ties can be inferred from co-location data, potentially compromising user anonymity [3].

However, large-scale datasets that include both mobility data and ground-truth social connections for the same individuals are extremely rare. This scarcity is partly due to privacy constraints limiting access to mobility data and partly because social network graphs and mobility records are rarely available for the same set of people. Consequently, only a small number of organizations can access both datasets, making it difficult to train deep models for relationship inference. Most existing methods[14, 25, 26, 30] either rely on supervised learning with explicit social connection labels or adopt unsupervised approaches based on manually defined features or location semantics, thereby limiting their generalizability. Yet, as shown in [23, 24], social ties often hinge on meeting frequency and location popularity—both of which can be derived from raw trajectory data independently of the underlying geography. Despite this, many existing approaches remain tailored to the specific datasets on which they were trained, inhibiting their ability to generalize across different regions [16–18].

While various deep learning strategies—such as contrastive learning [4], transfer learning [12], and meta-learning [29]—have been explored to enhance the generalizability of human mobility models [7, 15, 35], to the best of our knowledge, no work has specifically addressed generalizability for social relationship inference from trajectory data.

Beyond model-based transfer learning, embedding space alignment—a foundational technique for the rapidly evolving field of multimodal learning—offers a more generalizable framework for various downstream models and tasks [13, 27].

**Figure 1:**
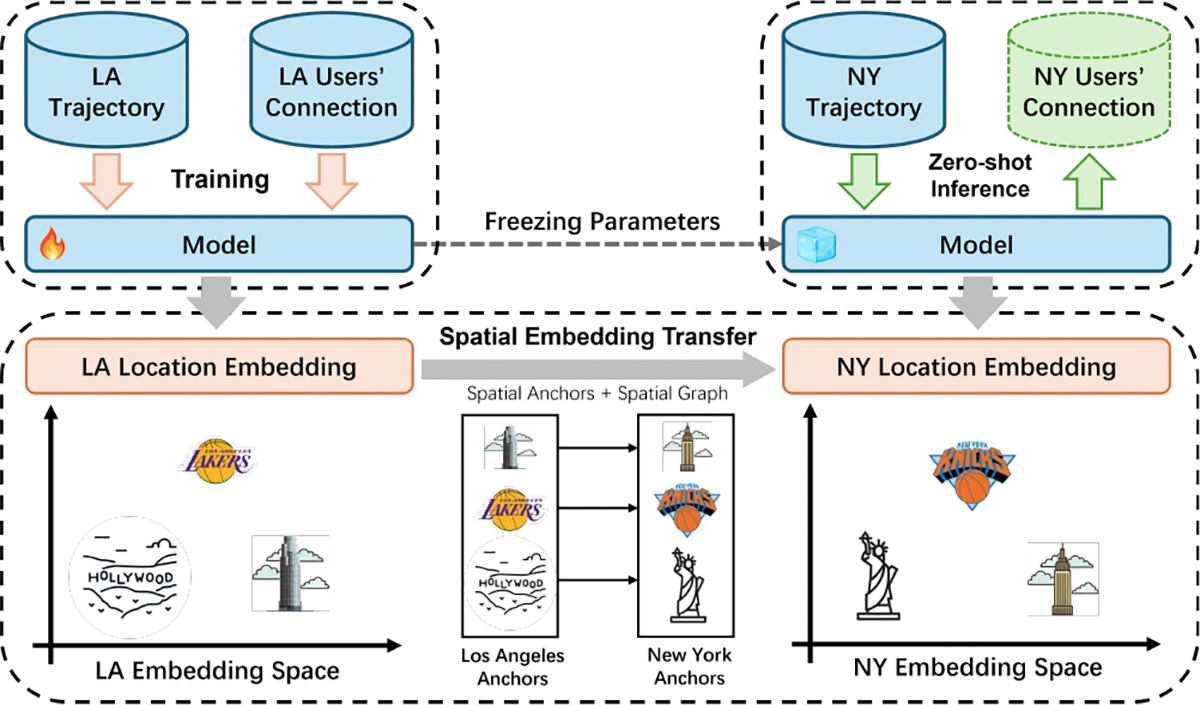
An illustration of how the spatial embedding transfer framework enables transferability across datasets.

Therefore, in this paper, we employ spatial embedding alignment to generalize social inference models. To illustrate our high-level idea, consider the example in Figure 1. First, a model is trained on a labeled dataset, termed a source dataset, following a standard supervised learning manner; in the figure, the source dataset is from Los Angeles (LA). To enable cross-dataset transferability, spatial embedding transfer methods are applied to align the location embedding distribution of a target dataset, New York (NY), in our example, with that of LA. Since the model is trained on LA’s embedding space, an effective embedding transfer method ensures that the transformed NY embeddings remain interpretable by the pre-trained LA model. Intuitively, landmarks in NY (e.g., the Statue of Liberty) are mapped to locations in the LA embedding space where similar landmarks (e.g., the Hollywood sign) exist. Since these landmarks have comparable popularity (and perhaps similar semantics for the purposes of social inference) in the two datasets, they are expected to contribute similarly to the inference of social ties. Our spatial embedding transfer enables pre-trained models to be transferable across different geographic regions by matching key spatial and contextual relationships in the location embedding spaces irrespective of the underlying geographic semantics.

To realize the above idea, we introduce a transferable social relationship inference framework that is initially trained on a source dataset (e.g., LA) and then generalized to a target dataset (e.g., NY) by aligning the target location embeddings with those of the source. Our framework comprises two main components—a Universal Social Relationship Classifier (USRC) model and a Spatial Embedding Transfer (SET) module—and is designed to enable zero-shot inference on target datasets without social relationship labels. USRC is a deep learning inference model that takes two trajectories—each belonging to a different individual—and predicts their social relationship. After USRC is trained on the source dataset, its location embedding layer serves as the “source embedding distribution” for transferability across datasets. Our SET module then aligns the location embedding features of target (unlabeled) datasets with this distribution.

Our key contribution lies in the alignment step, which integrates both SET and USRC to transfer knowledge across locations. First, SET identifies anchor points in LA and NY by matching locations based on the similarity of their ranked location popularity. For instance, NY’s most popular location is paired with LA’s, inheriting the pre-trained USRC embedding from LA. This reflects the insight that the likelihood of social connection depends on where people meet—co-visit at a popular landmark like the Empire State Building is generally less indicative of friendship than co-visit at a private residence.

Next, we feed sequences of NY embeddings, derived from user co-visits, pairwise and masked, into a USRC-based contrastive learning framework, freezing the USRC parameters from LA. The goal is to fine-tune the embeddings so they also capture co-visit patterns, leveraging USRC’s pre-trained knowledge from LA. In sum, this alignment transfers both the “popularity” and “co-visit” features of each location (captured through its embedding) from LA to NY.

Across five datasets, our framework demonstrates exceptional generalization ability. Notably, when trained on a richer source dataset and tested on a different target dataset, it outperforms a model trained and tested on target dataset itself—improving the evaluation metric by 14.4%. This underscores the value of leveraging more comprehensive training data for transfer learning.

Our contributions in this study can be summarized as follows:

- A transferable social relationship inference framework that infers social relationships between users based on their trajectory data and can perform zero-shot inference on unlabeled datasets.
- A novel spatial embedding transfer method. The method is designed to align location embedding distributions of two cities based on their human mobility patterns. The aligned embedding space is interpretable by the pre-trained model while preserving the unique characters of the target dataset.
- Through experiments on five real-world datasets, we demonstrated the strong generalizability of our social relationship inference model. Our SET module outperformed other embedding alignment baselines, and our zero-shot inference results also surpassed those of other unsupervised friendship inference models.

## 2 Related Work

### Social relationship inference:

Discovering real-world connections among people by analyzing their mobility data is a critical aspect of modern trajectory data mining. EBM [23, 24], among the earliest methods, introduced an entropy-based framework that uses Rényi entropy to capture co-visit patterns and Shannon entropy to capture location popularity. It showed that popularity and co-visit patterns alone are sufficient for revealing social ties, making additional location semantics unnecessary for improving relationship inference. Walk2friend [3] applies graph embedding techniques to infer social relationships. Followed by a series of graph-based methods such as MRGAN [25], Heter-GCN [30], SRINet [26], and HMGCL [17]. These methods generally combine social network and location graphs together to form a heterogeneous graph and apply graph neural networks (GNN) to learn the embedding features of each user and location. By computing the similarity between users, the relationship can be scored and inferred. However, these studies typically require evaluation datasets with ground truth friendship data to initiate their heterogeneous graph approaches, which significantly limits their generalizability.

### Transfer Learning:

Transfer learning encompasses a wide range of sub-fields, including domain adaptation [36], domain generalization [34], embedding alignment [1], and meta-learning [20]. Although each subfield has its own assumptions and focus areas, they all share the common goal of transferring knowledge from one domain or task to another. In the context of spatial data, which often suffers from uneven label distribution across regions, transferring knowledge from one area to another is a promising approach to address this imbalance and enable generalization to unseen regions.

Currently, most spatial transfer learning methods focus on domain adaptation and meta-learning. For example, AdaTM [36] and DastNet [28] utilize domain adaptation techniques to enhance predictions in regions with limited data. Similarly, CHAML [5] and MetaStore [20] apply metalearning strategies to tasks such as the next POI recommendation and the placement of the stores. While these methods are grounded in transfer learning principles, they still rely on labeled data from the target region. Furthermore, the models used in these tasks, such as traffic prediction and POI recommendation, are often auto-regressive, meaning the transferred knowledge serves more as an augmentation to existing models rather than enabling training a transferable model from scratch. In this research, we propose a novel spatial transfer learning framework based on embedding alignment. Our method does not require labeled data from the target region and supports various alignment techniques, making it flexible and broadly applicable.

### Embedding alignment:

Embedding space alignment plays key roles in tasks like cross-lingual word translation [1], knowledge graph entities alignment [32], and graph analytics [9]. The fundamental concept is to find a mapping function that gives the optimal matching solution to map one distribution to another [13]. Unsupervised learning methods are commonly used for alignment, with the Wasserstein Generative Adversarial Network (GAN) being one of the foundational approaches [2]. For graph alignment, WAlign [11] employs graph neural networks as graph encoders and uses a Multilayer Perceptron as the discriminator in a Wasserstein GAN to learn to estimate the Wasserstein distance between two distributions. Once the discriminator is trained, it guides the adversarial training of the graph encoders, enabling them to map the target distribution to the source distribution effectively. SANA improves the method by involving the graph augmentation methods in the training process [21]. HyperAlign also involves the graph augmentation method and introduces contrastive learning to obtain more compatible graph structures [9].

Unlike well-studied graph alignment and cross-lingual word alignment algorithms, which are typically applied to target and source datasets with ground truth alignment labels, alignment between different geographical regions lacks such labels. As a result, it must be learned in a fully unsupervised manner, and its effectiveness can only be evaluated through the performance of downstream tasks. For spatial alignment, Takahiro et al. propose a spatial embedding alignment method based on anchor points. The hierarchical batch anchoring method is proposed to select anchor points according to the total number of visits in each urban area, and they then utilize an affine transformation matrix to learn the mapping function between distributions [31]. However, their anchor-based alignment relies solely on popularity, ignoring other insights from trajectory data. Moreover, affine transformation only guarantees alignment among anchor points, leaving its generalization to non-anchor points uncertain.

## 3 Preliminary

### Trajectory:

In this paper, we denote the trajectory of a user u in length l as a sequence of visit points ${Traj}_{u}={\left\{pt_{i}\right\}}_{i=1}^{l}$, where $pt_{i}=(loc,t)$, with loc is represented by a place id and t indicates the timestamp of the visit.

### Co-visit sequence:

When two users meet at the same place and time, i.e., their trajectories intersect at a set of points $pt_{i}$, we represent the sequence of these intersections as their co-visit sequence, the time threshold of the intersection is denoted as τ. We can formulate the co-visit sequence of user n and user m in length l as $CV_{n,m}={\left\{pt_{i}|pt_{i}.loc\in {Traj}_{u_{m}},\left|pt_{i}.t_{u_{n}}-pt_{i}.t_{u_{m}}\right|\leq \tau \right\}}_{i=1}^{l}$.

### Co-location sequence:

Beyond shared visits at the same time, individuals who visit the same location at different times can also indicate a potential social connection. In [8], this is referred to as a “followship” relationship, where one user’s visit influences the other to go to the same location later. Therefore, we formulate the co-location sequence of user n and user m in length l as $CL_{n,m}={\left\{{\left(loc,f_{n},f_{m}\right)}_{i}|{loc}_{i}\in {Traj}_{u_{n}},{loc}_{i}\in {Traj}_{u_{m}}\right\}}_{i=1}^{l}$, where $f_{n}=Count\left({loc}_{i},{Traj}_{u_{n}}\right)$ denote the visit frequency of ${loc}_{i}$ of user n.

### Source and Target dataset:

This research focuses on creating a generalizable method for inferring social relationships by training a model on one (source) dataset and applying it to another (target) dataset. The *labeled* dataset used for training is referred to as the **source**, denoted as $D_{S}$, while the *unlabeled* dataset to which the model is later applied is called the **target**, denoted as the $D_{T}$. Accordingly, the corresponding embedding spaces are termed the source and target location embedding spaces, respectively.

### Social relationship inference:

The social relationship inference based on trajectory data is defined as follows. We are given a set of N users, each associated with a trajectory ${Traj}_{u_{i}}$ in a dataset $D={\left\{{Traj}_{u_{i}}\right\}}_{i=1}^{N}$. We also have a social connection matrix $Y_{N\times N}\in \{0,\;1\}$ where $Y_{n,m}=1$ indicates that users $u_{n}$ and $u_{m}$ are friends, and $Y_{n,m}=0$ otherwise. The task is to learn a inference function $ℱ\left({Traj}_{u_{n}},{Traj}_{u_{m}}\right)\to {\overset{ˆ}{y}}_{n,m}$, which takes the trajectories of two users as input and outputs a prediction ${\overset{ˆ}{y}}_{n.m}$ indicating the social connectivity of two people.

### Transferable social relationship inference:

Suppose we have two datasets, S and T, each containing user trajectories, denoted as $D_{S}$ and $D_{T}$. We also have a social network matrix $Y_{S}$ for dataset S that indicates which users are friends. However, no such ground-truth social network information is available for dataset T. The goal is to leverage the labeled dataset (with $D_{S}$ and $Y_{S}$) to infer the social network $Y_{T}$ in dataset T (where $D_{T}$ only is provided).

Our Approach is to first learn a model ${ℱ}_{S}$ that maps from trajectories in $D_{S}$ to social connections: ${ℱ}_{S}:D_{S}\to Y_{S}$. Next, we fine-tune the trained model ${ℱ}_{S}$ on the unlabeled trajectories $D_{T}$ to obtain a new model ${\bar{ℱ}}_{T}$. Finally, we apply ${\bar{ℱ}}_{T}$ to $D_{T}$ to predict the social network ${\overset{ˆ}{Y}}_{T}.\;{\bar{ℱ}}_{T}:D_{T}\to {\overset{ˆ}{Y}}_{T}$ where ${\overset{ˆ}{Y}}_{T}$ represents the inferred social connections among users in the dataset T.

## 4 Methodology

In this section, we first propose a social connection inference model named Universal Social Relationship Classifier (USRC) and then propose the Spatial Embedding Transfer (SET) module to make USRC transferable across different cities and datasets.

### 4.1 Universal Social Relationship Classifier

To make the model transferable across different regions, we design a model that only uses location data to infer the social relationship between two users.

Firstly, the model takes the trajectory of user n and m as input, then the model preprocesses their trajectories to extract the Co-visit sequence and Co-location sequence of two users. After that we employed three independent encoders to learn from their trajectories, co-visit and co-location. The USRC model can be formulated as follows,

(1) $$USRC\left({Traj}_{u_{n}},{Traj}_{u_{m}},CL_{n,m},CV_{n,m}\right)\to {\overset{ˆ}{y}}_{n,m}$$

Before learning from the sequence data, an embedding module is employed to map location IDs into a continuous feature space.

(2) $$e_{i}=Emb\left({loc}_{i}\right)$$

(3) $$Emb\left({loc}_{i}\right)=\theta_{Emb}^{T}\cdot onehot\left({loc}_{i}\right)$$

where $loc_{i}$ denotes the location id to be embedded, $e_{i}$ denotes the feature representation of location i. onehot is the one-hot function, which maps the location id to a one-hot representation $onehot\left(loc_{i}\right)\in R^{\left|l\right|}$, where |l| is the number of locations in the current dataset. $\theta_{Emb}\in R^{|l|\times d}$ is the trainable parameter of the location embedding function, where d is the location embedding feature dimension. The location embedding function is trainable by downstream tasks, which makes the embedding feature of each location contains both the essential information of the current location (e.g., its popularity) and relationships with other locations.

After mapping the locations into the feature space, we can use a deep neural network to learn from a sequence of locations. Since the order of co-visit and co-location locations does not matter, we employ two Transformer Encoder networks without positional encoding to encode $CL_{n,m}$ and $CV_{n,m}$ and one additional Transformer Encoder to encode trajectories of two users.

(4) $$cv=max\left\|{Trans}_{cv}\left(Emb\left(CV_{n,m}\right)\right)\right\|$$

(5) $$cl=mean\left\|{Trans}_{cl}\left(Emb\left(CL_{n,m}\right)\right)\right\|$$

(6) $${traj}_{n}=mean\left\|{Trans}_{traj}\left(Emb\left({Traj}_{u_{n}}\right)\right)\right\|$$

(7) $${traj}_{m}=mean\left\|{Trans}_{traj}\left(Emb\left({Traj}_{u_{m}}\right)\right)\right\|$$

(8) $${\overset{ˆ}{y}}_{n,m}=\theta_{y}\left(\theta_{cl}cl+\theta_{cv}cv+\theta_{t}traj_{n}+\theta_{t}traj_{m}\right)$$

where ${Trans}_{cl},\;{Trans}_{cv}$ and ${Trans}_{traj}$ denote the Transformer Encoder for co-location, co-visit and trajectory, respectively, $cl\in R^{d},\;cv\in R^{d}$ and $traj_{n}\in R^{d}$ denote the feature representation of the co-location, co-visit sequence and trajectory of user n. $\theta_{cl}\in R^{d^{\prime }\times d},\;\theta_{cv}\in R^{d^{\prime }\times d},\;\theta_{t}\in R^{d^{\prime }\times d},\;\theta_{y}\in R^{1\times d^{\prime }}$ denote the weighting parameters of the model. The co-visit sequence between two users captures the most critical information about their social relationships. Therefore, we apply max pooling to extract the most salient features from the co-visit sequence for representation. On the other hand, while the co-location information and trajectory of each user serve as valuable supplements to co-visit data, especially given the general sparsity of co-visit data, the co-location and user trajectory sequence often contains irrelevant and redundant information. To address this, we employ mean pooling to aggregate and smooth the information within both the co-location sequence and trajectories of two users.

After modeling the co-visit, co-location, and trajectories of two users, we can now have a feature to represent the social relationship of two users ${\overset{ˆ}{y}}_{n,m}$. According to the label data that we have in the training dataset, we have $y_{n,m}\in \{0,\;1\}$. We use an MSE loss function, treating social connection inference as a regression problem. Predictions ${\overset{ˆ}{y}}_{n,m}$ near 1 indicate a higher likelihood of connection, while values near 0 suggest the opposite. Because there’s no need to make a binary classification for evaluation, no threshold is required. Moreover, the smooth gradients from MSE help the frozen model adapt more effectively during the following contrastive learning.

Incorporating the temporal information (e.g., time or duration) for each co-visit would increase the complexity of the social inference model and complicate subsequent transfer learning. We did not include temporal info in the USRC, as its impact is suspected to be low.

### 4.2 Spatial Embedding Transfer (SET)

**Figure 2:**
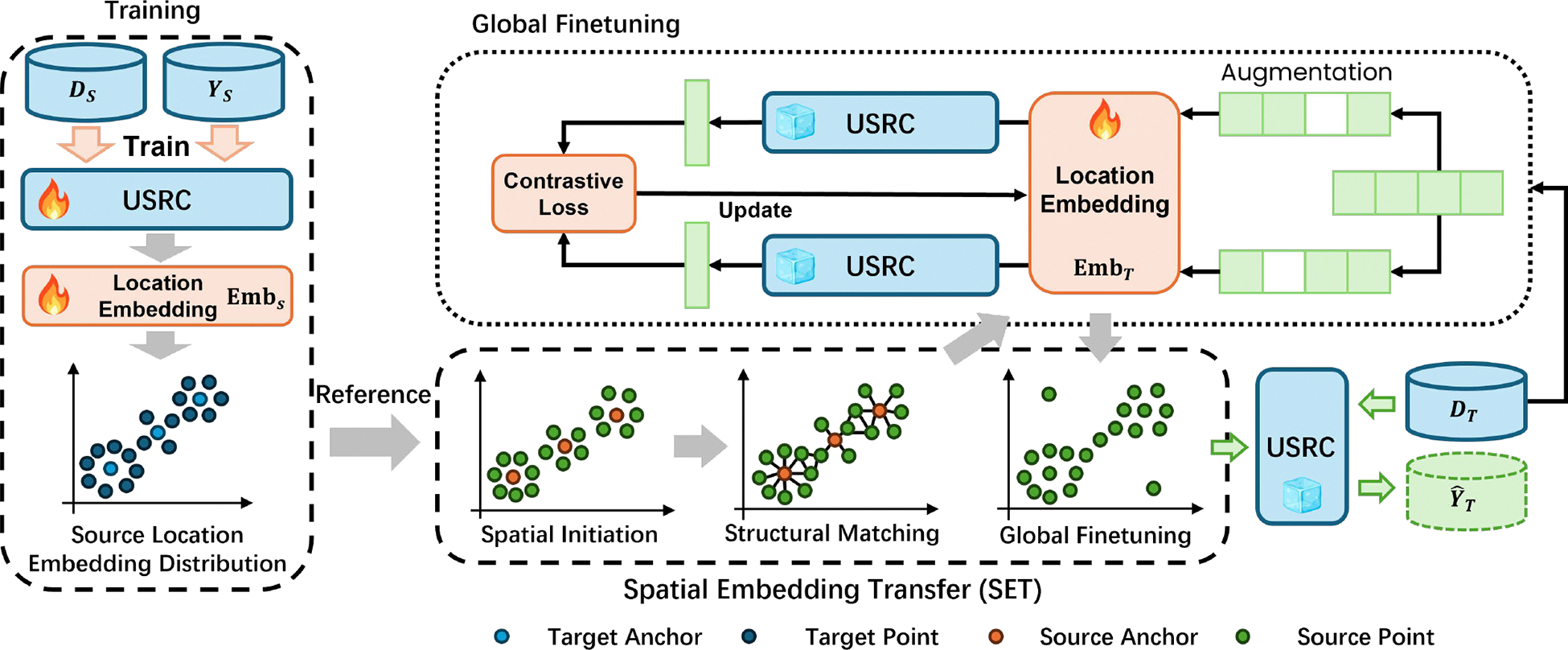
An illustration of the Spatial Embedding Transfer (SET) module.

To enable USRC to transfer across datasets, we first freeze all its parameters once training on source data is complete. We then use our SET module to align the target location embeddings with those of the source, and finally replace USRC’s frozen location embedding layer with these newly aligned embeddings. The key to transferability is how to map the embedding feature distribution of the target dataset to the source dataset properly. The SET module not only needs to align the target embedding distribution to the source space to be compatible with the frozen USRC, but it also needs to capture the unique co-location/visit patterns shown in the target dataset in a way the USRC can interpret. We propose the SET module to solve these two challenges in three steps: Spatial Initiation, Structural Matching, and Global Finetuning. The first two steps adapt the source embeddings to capture location popularity from the target dataset, while the final step incorporates the target’s co-location and visit patterns. The framework of the module is shown in Figure 2.

#### Spatial Initiation:

Instead of randomly initiating the location embedding of the target dataset, we propose a Spatial Initiation method to meet the assumption required by our Structural Matching method. We define spatial anchor points for this purpose. Prior research shows that different cities share similar visit-frequency patterns, meaning their most and least popular places maintain a consistent proportion across cities [31]. Therefore, sampling locations from two popularity distributions and pairing them as anchor points can effectively capture the structural similarities between cities. We use location entropy to compute popularity [22, 24]. By linear sampling from the ranked location entropy distribution, anchor points of each city are automatically selected. After pairing the ordered anchor points of the source and target cities, we obtain the correspondence relationship of location popularity between the two cities.

(9) $$E_{l}=-\sum_{u,P_{u,l}\neq 0}P_{u,l}logP_{u,l}$$

10) $${Anchor}_{S}\overset{p}{∼}Rank\left(E_{l_{S}}\right)$$

(11) $${Anchor}_{T}\overset{p}{∼}Rank\left(E_{l_{T}}\right)$$

(12) $${Anchor}_{T\to S}=zip\left({Anchor}_{T},{Anchor}_{S}\right)$$

where $P_{u,l}$ denotes the probability that a randomly picked check-in at location l belongs to user u. $Rank\left(E_{l_{S}}\right),\;Rank\left(E_{l_{T}}\right)$ denote the ranked location entropy of source and target datasets S and T, respectively. $p=\alpha \cdot min\left(\left|l_{S}\right|,\left|l_{T}\right|\right)$ denotes the number of locations sampled as anchor points; it represents α percent of the locations from the dataset with fewer locations. ${Anchor}_{S}$ and ${Anchor}_{T}$ denote the anchor points set of datasets S and T, which are sampled sequentially in linear order based on their location entropy distributions. By pairing the corresponding anchor points between the two datasets, we obtain the dictionary ${Anchor}_{T\to S}$. The matched anchor points between two datasets indicate which places share similar popularity. We then use this information to initialize $Emb_{T}$ by matching each location in the target dataset to a comparable location in the source dataset.

(13) $${Emb}_{T}\left[{loc}_{i}\in {Anchor}_{t}\right]={Emb}_{S}\left[{Anchor}_{T\to S}\left[{loc}_{i}\right]\right]$$

Most of the time, the number of locations in two different datasets does not match exactly, and we also want to maintain flexibility during this phase. This means we don’t want our initialized target embedding space to be exactly the same as the source one. Therefore, we select only a subset of locations as anchors and set the embeddings of non-anchor locations according to the following rules.

(14) $${Emb}_{T}\left[{loc}_{i}\notin {Anchor}_{t}\right]=\;{Emb}_{S}\left[{Anchor}_{T\to S}\left[arg\;min\;dis\left({loc}_{i},{Anchor}_{T}\|{Graph}_{T}\right)\right]\right]+\delta$$

For each place that is not selected as an anchor in the initiation process, the arg min dis function finds its closest anchor point according to ${Graph}_{T}$ distance and assigns its embedding according to that anchor point. Random noise δ∼𝒩(0,σ) is added to their embeddings to enhance diversity and promote regularization. ${Graph}_{T}$ is essentially the OD (Origin-Destination) Matrix of T, a common tool in transportation planning, logistics, and urban mobility that shows the number of trips from each origin to each destination. Here, we derive these flows directly from trajectory data.

#### Structural Matching:

We propose a structural fine-tuning module to further align the target location embeddings with the source embedding space. Once each target location is assigned a value from the source distribution, $Emb_{T}$ occupies the same feature space as $Emb_{S}$. We then refine their alignment by incorporating OD flows derived from the trajectory data, ensuring a more precise matching of the two distributions. Algorithm 1 describes the Structural Matching approach to align target and source embedding distributions using the OD graph of the target dataset. In each iteration, the algorithm updates the embeddings for anchor points and their neighbors, guiding them toward the anchor point’s nearest feature in the source embedding $Emb_{S}$. The flow on the edges in the graph serve as weights, influencing how far each node moves. The learning rate decreases over iterations, allowing for a gradual adjustment of the target embedding distribution.

**Algorithm 1:** Structural Matching

**Input:** $Emb_{T},\;Emb_{S},\;{Anchor}_{T},\;{Graph}_{T}$, Number of iterations τ, Threshold ϵ, Learning rate *LR*	
**Output:** $Emb_{T}$	
1:	**for** each iter∈[1,τ] **do**
2:	**for** each ${loc}_{i}\in {Anchor}_{T}$ **do**
3:	$w=Emb_{S}\left[argmindis\left({loc}_{i},Emb_{S}\right)\right]$
4:	$lr=LR\cdot \left(1-\frac{iter}{\tau }\right)$
5:	**for** each $loc_{j}\in {Graph}_{T}\left[{loc}_{i}\right]$ **do**
6:	**if** ${Graph}_{T}\left[{loc}_{i},loc_{j}\right]>\epsilon$ **then**
7:	${Emb}_{T}\left[loc_{j}\right]=lr\cdot {Graph}_{T}\left[loc_{i},loc_{j}\right]\cdot \left(Emb_{T}\left[loc_{j}\right]-\right.w)$
8:	**end if**
9:	**end for**
10:	**end for**
11:	**end for**
12:	**return** ${Emb}_{T}$

#### Global Finetuning:

After Structural Matching, we have aligned the location embedding distribution of the target dataset to the embedding space on which the USRC has been trained. However, each city or dataset has unique human mobility patterns, and these unique characters often play an important role in location representation. In this section, we propose an unsupervised location embedding finetuning method, which aims to finetune the target location embedding to learn the unique co-location/visit pattern of the target dataset and make it a better fit for the pre-trained model.

The source embedding distribution is trained using gradients passed from the USRC model, making the USRC the only model capable of interpreting the distribution. Therefore, to ensure that the fine-tuned target embedding remains suitable for the pre-trained model, the pre-trained USRC must be involved in the fine-tuning process.

Towards this end, we propose an embedding finetuning method based on contrastive learning. The pre-trained USRC takes augmented user co-location and co-visit sequences as input and outputs hidden representations of these augmented pairs. We only augment co-visit and co-location sequences and mask the trajectory embeddings to ensure the model zeroes in on the target dataset’s unique co-location patterns—critical for social connection inference. Full trajectories contain excessive redundant information that isn’t needed for this task. According to the similarity between output representations, location embeddings are trained to fit in the input space of USRC. The formal definition of the algorithm is as follows. Firstly, we define our location sequence augmentation function,

(15) $$Aug({\left\{{loc}_{i}\right\}}_{i=1}^{l})={\left\{{loc}_{i}\neq {loc}_{k}\right\}}_{i=1}^{l-1}$$

which randomly removes $loc_{k}$ from $loc_{\left(1,l\right)}$. The augmentation aims to slightly modify a co-location or co-visit sequence by randomly removing one of the locations in the sequence. We only augment sufficiently long sequences where omitting a single location won’t alter their overall meaning, ensuring that most of the original information is preserved. In Algorithm 2, we first replace the location embedding layer of the pre-trained USRC with the embedding layer of the target dataset, to which we have applied Spatial Initiation and Structural Matching. Subsequently, we generate a contrastive learning pair using the augmentation function to the co-location or co-visit sequence in a training sample. USRC is employed to create the feature representation of the current sample. Next, we compute the contrastive loss, which aims to maximize the cosine similarity between augmented samples derived from the same co-location/visit pair while minimizing the similarity for negative pairs within the same batch. The gradient of the contrastive loss is used to update the embedding layer of USRC, specifically the location embedding corresponding to the target dataset.

**Algorithm 2:** Global Matching

**Input:** ${Emb}_{T}$, Pretrained USRC, Target Dataset $D_{T}$, Iteration τ, Batch size *bs*, Temperature t, Learning rate *lr*	
**Output:** ${Emb}_{T}$	
1:	$USRC.Emb\;\leftarrow {Emb}_{T}$
2:	**for** each iter∈[1,τ] **do**
3:	sample minibatch ${\left\{\left({Traj}_{u_{n}},{Traj}_{u_{m}},{CL}_{n,m},{CV}_{n,m}\right)\right\}}_{i=1}^{bs}$
4:	**for** each k∈[1,bs] **do**
5:	$h_{2k-1}=USRC\left({\left\{{Traj}_{u_{n}},{Traj}_{u_{m}},Aug\left({CL}_{n,m}\right),Aug\left({CV}_{n,m}\right)\right\}}_{k}\right)$
6:	$h_{2k}=USRC\left({\left\{{Traj}_{u_{n}},{Traj}_{u_{m}},Aug\left({CL}_{n,m}\right),Aug\left({CV}_{n,m}\right)\right\}}_{k}\right)$
7:	**end for**
8:	**for** each i∈[1,2bs] and j∈[1,2bs] **do**
9:	$s_{ij}=\frac{h_{i}^{⊤}h_{j}}{\left\|h_{i}\right\|\cdot \left\|h_{j}\right\|}$
10:	**end for**
11:	$l(i,j)=-log\left(\frac{exp\left(s_{ij}/t\right)}{\sum_{k=1,k\neq i}^{2bs}exp\left(s_{ik}/t\right)}\right)$
12:	$\text{Loss}=\frac{1}{2bs}\sum_{k=1}^{bs}\;[l(2k-1,2k)+l(2k,2k-1)]$
13:	$\underset{\text{USRC.Emb}}{min}\;Loss\left(USRC.Emb,\left.D_{T}\right)\right.$
14:	**end for**
15:	**return** ${Emb}_{T}=\;USRC.Emb$

Notably, while the training loss flows through USRC’s parameters, they remain unchanged, preserving the model’s source-domain knowledge. At the same time, the loss updates the embedding layer, teaching USRC to interpret the revised location embeddings. Consequently, the location embedding is fine-tuned into a distribution fully compatible with USRC.

## 5 Experiments

### 5.1 Datasets

For our experiments, we use two real-world publicly available user check-in datasets with labeled friendships, Gowalla and Foursquare [6, 33]. Users with check-in data and there friendship relationships in Los Angeles, New York and Stockholm(ST) are extracted from the datasets, forming five independent datasets. We then infer the relationships across all users within the same dataset.

To refine the quality of the dataset, consistent with prior studies using these datasets [24], we assumed that users without any co-location are unknown to each other and removed those user pairs from the dataset. Table 1 depicts the key statistics for each dataset. Owing to distinct check-in data collection methods, Gowalla and Foursquare differ significantly, even within the same city—justifying our treatment of them as independent datasets.

### 5.2 Baselines

We first compare the zero-shot inference performance of our method with other unsupervised social relationship inference baselines. Next, we evaluate our spatial embedding transfer method against other embedding alignment approaches.

#### Unsupervised inference baselines:

The following baselines do not require label data for their evaluation. To the best of our knowledge, there are few unsupervised friendship inference models at this stage; the included two baselines are the most recent ones we could find.

##### Walk2friend [3]:

Walk2Friend constructs a heterogeneous graph of users and locations. Through random walk and skip-gram embedding algorithms, it obtains user embeddings and infers relationships between pairs of users by computing their cosine similarities.

##### Heter-GCN [30]:

Heter-GCN also relies on a heterogeneous graph structure, using an unsupervised loss to train a graph convolutional network that captures relationships among both heterogeneous and homogeneous nodes. The inference stage then compares user node embeddings to determine their similarity.

#### Embedding alignment baselines:

We compare our SET module with other embedding alignment methods by evaluating how well the inference model uses each alignment for zero-shot prediction.

##### HBP [31]:

HBP applies a hierarchical batch anchoring method to select anchor points and then applies Procrustes alignment to compute the rotational matrix, which could map the target embedding distribution to that of the source.

##### HBA [31]:

HBA applies the same anchoring method of HBP but uses the Affine alignment to align two distributions.

##### LSNA [19]:

LSNA is a social network alignment method that utilizes graph convolution layers to learn structural information and then uses cross-network convolution to generate the aligned features of nodes in two graphs.

##### SANA [21]:

SANA uses Graph Attention Network as the encoder and utilizes graph augmentation to refine the alignment relationship between nodes of different graphs.

##### WAlign [11]:

WAlign uses a GCN to encode graphs and then applies Wasserstein GAN (WGAN) to train a generator to map the target distribution to the source distribution.

##### HyperAlign [9]:

HyperAlign employs contrastive learning in graph embedding and uses graph augmentation to create hyperedges to augment the topological relationship. The alignment network is also trained based on WGAN.

LSNA, SANA, and HyperGAN are network alignment methods designed to find the optimal alignment solution for each individual node in the target and source graphs. That is, the model identifies which node in the source graph is most similar to a given node in the target graph, considering the graph structure and node features. In our experiments, we used these methods to determine node-to-node alignment solutions. Subsequently, we assigned the embedding features of the nodes in the source embedding layer to their corresponding nodes in the target layer. HBP, HBA, SET, WAlign, and our method SET belong to embedding alignment methods, which aim to find a mapping function to directly map the target embedding distribution to that of the source.

### 5.3 Experiment Setting

In our experiments, we trained five USRC models, each on a different dataset, and evaluated each model on its own validation set corresponding to the same training dataset. Thus, these results represent the upper bound of USRC’s performance for that dataset under a fully supervised setup. Subsequently, for each model, we apply various embedding alignment methods to map the other four target datasets’ embeddings onto the source embedding space where the model was initially trained. The evaluation is based on the model’s performance on the transferred datasets. We average each dataset’s transfer performance from the other four to compare our zero-shot inference performance with other unsupervised friendship inference methods.

The embedding dimension for location embedding layers for all datasets is set as 256. σ is set as 0.5. For each dataset, the top 500 locations with the highest location entropy are selected as anchor points, followed by a linear sampling of 60% from the remaining locations. The Structural Matching learning rate is set as 0.1, and the number of iterations is set as 10. The global Global Finetuning learning rate is set as 0.001, the batch size is set as 4, and the number of iterations is set as 3.

### 5.4 Evaluation Metrics

To evaluate the prediction performance of all models, we employed two widely used metrics in social relationship inference: the Area Under the Precision-Recall Curve (PR-AUC) and the Area Under the Receiver Operating Characteristic Curve (ROC-AUC). Because the dataset has far more non-friend pairs than friend pairs, thus a significant class imbalance in the dataset, we use PR-AUC as our primary metric to evaluate performance across different datasets.

### 5.5 Experiment Results

In this section, we present the transfer inference result of our method, compare the result with other unsupervised social relationship inference models, and then compare our SET module with other spatial embedding transfer baselines.

Firstly, we present the transfer inference results of our method. Table 2 presents a matrix of PR-AUC results for our model across five datasets. Each row represents the dataset used for training, and each column indicates the dataset used for inference. The diagonal cells with underlines (where the model is trained and tested on the same dataset) correspond to the supervised scenario, while the off-diagonal cells show the zero-shot transfer results (i.e., the model is trained on one dataset but used for inference on another). For example, in the 1st column of Table 2, the 1st cell shows the performance of a USRC model trained and evaluated in a supervised manner on the Gowalla_LA dataset. The 2nd, 3rd, 4th, and 5th cells show the transfer result of our proposed framework: the SET module is applied to adapt the same USRC model, trained on Gowalla_LA, to the Gowalla_NY, Gowalla_ST, Foursquare_LA, Foursquare_NY datasets.

As shown in Table 2, our approach provides outstanding transferability across both different cities and datasets. Notably, when trained on a high-quality dataset like Gowalla_LA, it even outperforms the best supervised results on the Foursquare_LA and Foursquare_NY datasets.

Next, we compare our zero-shot inference results against other unsupervised inference baselines. For our method, we report the average zero-shot performance on each dataset, where the model has been transferred from each of the remaining four datasets. As shown in Table 3, our method significantly outperforms other baselines. The performance of walk2friend is highly dependent on random walks, which become inefficient on large datasets, leading to weak results. Heter-GCN relies on positive pairs selected through manually crafted features, causing poor generalization across various datasets. In contrast, our SET module automatically adapts to new datasets without predefined rules. Meanwhile, it avoids the computational overhead of graph embedding methods, resulting in better scalability. Finally, we compare our SET module to other embedding transfer methods. All approaches use the same transfer framework and the same pre-trained USRC model, so the focus here is on the alignment quality of the embedding space. Table 4 reports each model’s average PR-AUC and ROC-AUC performance, using the dataset indicated in the table’s column for training. The values shown represent the average zero-shot results across the remaining four datasets for each column.

Table 4 shows that our transfer method consistently outperforms all other embedding alignment baselines across every dataset, and it is the only approach that achieves performance on par with supervised methods. Additionally, we observe that embedding mapping strategies (HBP, HBA, WAlign) generally outperform network alignment techniques (LSNA, SANA, HyperGAN), suggesting that an exact match between target and source distributions is not necessary; a more flexible mapping function can yield a more suitable target distribution.

The spatial and temporal granularity of human mobility datasets, as well as the quality of the social relationship network, are two important factors that influence the performance of the proposed transfer method. The Foursquare dataset has a much denser check-in frequency than Gowalla, therefore, the overall transfer performance between these two datasets is lower than that achieved within the same dataset across different cities. Meanwhile, the Gowalla_ST dataset contains a dense and high-quality social relationship network, resulting in the best transfer performance when used as either the target or the source city.

### 5.6 Ablation Study

Table 5 presents an ablation study of our SET module components:

- Random – The USRC frozen model uses completely random location embeddings.
- Structural – The Global Finetuning module is removed, so embeddings rely only on Spatial Initiation and Structural Matching, capturing location popularity but not co-location/visit information.
- Global – Instead of using embeddings from Structural Matching, a randomly initialized layer is passed into the Global Finetuning module—representing the opposite scenario in which co-location/visit information is prioritized while location popularity alignment is omitted.

As observed from Table 5, models that take random embeddings as location features cannot perform well in social relationship inference because random embeddings cannot capture location information, such as popularity and connectivity. Therefore, although the frequency of co-visits and co-location contains information about whether two people are friends, where people meet plays a more critical role in inferring their social connectivity. Another observation is that both Structural Matching and Global Finetuning can successfully align location embeddings to the source space. This indicates that both location information (e.g., popularity, connectivity with other locations) and co-location patterns are important for social relationship inference. The high-quality alignment of SET module is achieved by integrating information from both aspects. Structural Matching provides fundamental location information, offering a suitable initialization for Global Finetuning. After the initialized embeddings capture essential mobility patterns shared across datasets, Global Finetuning refines the alignment by learning unique co-location patterns specific to the dataset, achieving the best matching.

### 5.7 Parameter Sensitivity

**Figure 3:**
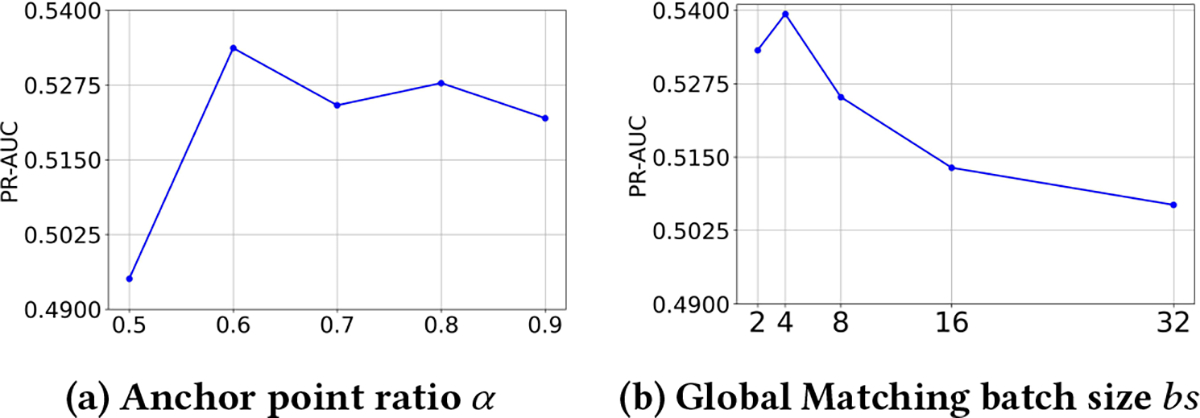
Overall model performance on different hyperparameter settings.

To analyze the effects of hyperparameters of the SET, we use a USRC model trained on the Gowalla_LA dataset and transfer it to other datasets with different parameter settings. The results are compared by measuring the average performance of the model on the other datasets. We analyze two important hyperparameters of the SET: α, the ratio of locations selected as anchor points during Spatial Initiation; and bs, the batch size used during Global Matching. The results are shown in figure 3.

As we can see from Figure 3(a), the alignment quality does not continue to improve as the number of anchor points increases. The best performance is achieved when 60% of the locations are selected as anchors, while the remaining 40% remain flexible. The flexibility preserved during the Spatial Initiation supports our assumption that different datasets have different embedding distributions and improves the effectiveness of the subsequent Structural and Global Matching.

As shown in Figure 3(b), the Global Matching module produces the most distinguishable location embedding distribution when the batch size is 4. In contrastive learning, a larger batch size typically provides more negative samples, which encourages the model to learn more generalizable embeddings. However, our objective is not to learn general-purpose representations, but rather to fine-tune the model to closely align with the specific embedding distribution of the pretrained USRC model. A smaller batch size facilitates this alignment by limiting the diversity of negative samples, allowing the model to focus on fine-grained distinctions that are more relevant to the USRC model-specific embedding space. In particular, it encourages the model to concentrate on matching the distributional characteristics of the pretrained embeddings rather than being regularized by a large number of uninformative negatives. Consequently, this leads to more specialized and discriminative representations tailored to our target task.

### 5.8 Computational Complexity

In this section, we analyze the computational complexity of SET. The complexity of Spatial Initiation is $O\left(NL+L\log L+L^{2}\;log\;L\right)$, where N denotes the number of users, and L denotes the number of locations. The complexity for Structural Matching is $O\left(\tau kL^{2}\right)$, where k denotes the average number of neighbors of a location in OD graph. The complexity of Global Matching is $O\left(bs^{2}N\right)$. Since the number of epochs τ and the batch size bs are generally small compared to the number of users and locations, they can be considered constants in this analysis. Therefore, the overall complexity is: $O\left(NL+L\log L+L^{2}\;log\;L+kL^{2}+N\right)$. Given that the alignment process is a one-time cost and eliminates the need to train a new friendship inference model, the time cost of the SET module is acceptable, especially compared to the time required to train a model from scratch.

We also consider the computational resources required during alignment. Graph-based embedding alignment methods, including LSNA, SANA, WAlign and HyperAlign, typically require the entire graph to be loaded into GPU memory for computation. From the dataset statistics, we know that the size of such a graph is $L^{2}$. Since the number of locations L is typically large for most datasets, the enormous memory cost of the backward propagation renders these methods difficult to use in practice. In our experiments, we found that it was not feasible to run these methods on a GPU with 80 GB of memory for the Foursquare_NY dataset, which has 33,128 locations. In contrast, our method does not rely on full-graph computation. By breaking the alignment process into multiple steps, we significantly reduce memory requirements and achieve alignment with much lower GPU usage. In practice, the whole transfer process takes no more than 10 minutes on all experimental datasets.

## 6 Conclusion and Future Directions

In this paper, we proposed a transferable social relationship inference framework, which includes a trajectory-based social relationship inference model (USRC) and a spatial embedding transfer method (SET). The spatial embedding transfer is conducted in an unsupervised manner by leveraging information extracted from trajectory data. It maps the location embedding distribution of the target dataset to the source embedding space, which the relationship inference model has been trained on. This enables the model to transfer the knowledge it learned from a labeled dataset to unlabeled ones. Different from other domain adaptation methods that assume embeddings should be aligned perfectly, we build on our assumption that each city could have its own feature space distribution. This unique distribution can be learned from the human mobility pattern of the dataset. By leveraging the power of contrastive learning, we can approximate this distribution and boost the performance of the downstream model. Through experiments on five publicly available real-world datasets, our method demonstrates exceptional transferability. Notably, the zero-shot inference performance of the model, when trained on a high-quality dataset, surpasses that of the supervised inference methods. Our findings serve as a promising step toward the development of a geospatial foundation model, one that can be trained on large-scale, high-quality data while remaining highly generalizable across a broad range of datasets and downstream tasks.

Due to the sensitivity of location data and social relationship networks, current research is constrained by the limited availability of datasets with diverse granularities, such as CDR data or datasets including the specific type of social relationships (e.g., colleagues, friends, family). Exploring such datasets would be a valuable direction for future research. Moreover, incorporating richer contextual information into spatial transfer learning could enable modeling the correlation between spatial context and human mobility patterns, offering another promising research direction.

## Figures and Tables

**Table 1:** Statistics of the datasets used

Dataset	Gowalla_LA	Gowalla_NY	Gowalla_ST	Foursquare_LA	Foursquare_NY

# users	7,498	7,302	10,693	5,736	9,199
# locations	10,146	10,866	23,471	20,166	33,128
# check-ins	367,586	368,027	686,851	1,189,571	1,881,703
# friends	2,270	2,424	6,101	936	1,896
# un-friends	60,893	71,304	104,673	41,370	95,100

**Table 2:** Cross-dataset PR-AUC results of our transferable social relationship inference model.

PR-AUC		Evaluation datasets				
		Gowalla_LA	Gowalla_NY	Gowalla_ST	Foursquare_LA	Foursquare_NY

	Gowalla_LA	**0.6548**	0.5270	0.7792	**0.5253**	**0.5677**
	Gowalla_NY	0.5307	**0.5446**	0.6891	0.4122	0.3801
Training datasets	Gowalla_ST	0.5208	0.4193	**0.7961**	0.4466	0.5174
	Foursquare_LA	0.5834	0.4550	0.7302	0.5007	0.5414
	Foursquare_NY	0.5560	0.5122	0.6803	0.4739	0.4962

**Table 3:** Performance comparison of unsupervised social relationship inference models

Method	Gowalla_LA		Gowalla_NY		Gowalla_ST		Foursquare_LA		Foursquare_NY	
	PR-AUC	ROC-AUC	PR-AUC	ROC-AUC	PR-AUC	ROC-AUC	PR-AUC	ROC-AUC	PR-AUC	ROC-AUC

Walk2friend	0.1823	0.7125	0.1323	0.6741	0.2433	0.7306	0.1033	0.7718	0.1086	0.7457
Heter-GCN	0.3478	0.7728	0.2773	0.7619	0.3191	0.7155	0.2901	0.7913	0.3097	0.7835
OURS	**0.5567**	**0.8249**	**0.4980**	**0.8871**	**0.7197**	**0.7736**	**0.4704**	**0.8178**	**0.4964**	**0.8747**

**Table 4:** Zero-shot inference comparison of various spatial embedding transfer methods

Method	Gowalla_LA		Gowalla_NY		Gowalla_ST		Foursquare_LA		Foursquare_NY	
	PR-AUC	ROC-AUC	PR-AUC	ROC-AUC	PR-AUC	ROC-AUC	PR-AUC	ROC-AUC	PR-AUC	ROC-AUC

HBP	0.2470	0.6607	0.2614	0.6538	0.1659	0.5904	0.1477	0.5826	0.2108	0.6268
HBA	0.2864	0.7091	0.2539	0.6895	0.1492	0.5867	0.1382	0.5681	0.1928	0.6647
LSNA	0.2336	0.6567	0.3026	0.7131	0.1526	0.5882	0.1680	0.6399	0.1988	0.5917
SANA	0.1693	0.6271	0.1931	0.6415	0.1438	0.5653	0.1259	0.5293	0.1423	0.5638
WAlign	0.3323	0.7503	0.3417	0.7419	0.1541	0.5754	0.1606	0.5869	0.2315	0.6514
HyperGAN	0.2278	0.6672	0.2529	0.6738	0.2005	0.6314	0.2097	0.6561	0.2821	0.6868
SET	**0.5400**	**0.8640**	**0.4410**	**0.8154**	**0.4760**	**0.8055**	**0.5266**	**0.8332**	**0.5140**	**0.8254**

**Table 5:** Zero-shot inference comparison of variants of the SET module

Method	Gowalla_LA		Gowalla_NY		Gowalla_ST		Foursquare_LA		Foursquare_NY	
	PR-AUC	ROC-AUC	PR-AUC	ROC-AUC	PR-AUC	ROC-AUC	PR-AUC	ROC-AUC	PR-AUC	ROC-AUC

Random	0.1934	0.5953	0.1991	0.5805	0.1408	0.5599	0.1251	0.5463	0.1331	0.4969
Structural	0.2859	0.6843	0.2616	0.6551	0.1920	0.5982	0.3081	0.6906	0.2749	0.6734
Global	0.4381	0.8342	0.3831	0.7777	0.2283	0.6202	0.3211	0.6861	0.3068	0.7243
SET	**0.5400**	**0.8640**	**0.4410**	**0.8153**	**0.4760**	**0.8055**	**0.5266**	**0.8332**	**0.5140**	**0.8254**
